# Three-year outcomes of surgical valve replacement with Dafodil™ pericardial bioprosthesis: Dafodil™-1 trial

**DOI:** 10.3389/fcvm.2024.1393762

**Published:** 2024-05-30

**Authors:** Channabasavaraj Shivalingaiah Hiremath, Anil R. Jain, Anurag Garg, Atul A. Maslekar, Nirmal K. Gupta, Binay Krishna Sarkar, Seetharama Bhat, Manish Porwal, Zile Singh Meharwal, Yugal Kishore Mishra, Prashanth Vaijyanath, Vijay Grover, Shiv Kumar Chaudhary, Subash S. Rajput, Rajan Sethuratnam, Naman Shastri

**Affiliations:** ^1^ Department of Cardiothoracic and Vascular Surgery, Sri Madhusudan Sai Institute of Medical Sciences and Research, Sri Sathya Sai Sanjeevani Group of Hospitals, Sathya Sai Grama Muddenahalli, Chikkaballapura, India; ^2^ Department of Cardiovascular and Thoracic Surgery, EPIC Hospital, Ahmedabad, India; ^3^ Department of Cardiothoracic Surgery, Dr D. Y. Patil Medical College & Hospital, Pune, India; ^4^ Department of Cardiothoracic and Vascular Surgery, Narayana Multispeciality Hospital, Ahmedabad, India; ^5^ Department of Cardiovascular and Thoracic Surgery, Sanjay Gandhi Postgraduate Institute of Medical Sciences, Lucknow, India; ^6^ Department of Cardiothoracic and Vascular Surgery, Nil Ratan Sircar Medical College and Hospital, Kolkata, India; ^7^ Department of Cardiothoracic and Vascular Surgery, Sri Jayadeva Institute of Cardiovascular Sciences & Research, Bangalore, India; ^8^ Department of Cardiothoracic Surgery, Convenient Hospitals Limited, Indore, India; ^9^ Department of Cardiothoracic and Vascular Surgery, Fortis Escorts Heart Institute, New Delhi, India; ^10^ Department of Cardiothoracic and Vascular Surgery, Manipal Hospital, New Delhi, India; ^11^ Department of Cardiothoracic Surgery, Kovai Medical College and Hospital, Coimbatore, India; ^12^ Department of Cardiothoracic and Vascular Surgery, Dr. Ram Manohar Lohia Hospital, New Delhi, India; ^13^ Department of Cardiothoracic and Vascular Surgery, All India Institute of Medical Sciences (AIIMS), New Delhi, India; ^14^ Department of Cardiothoracic and Vascular Surgery, Ram Manohar Lohia Hospital, Lucknow, India; ^15^ Department of Cardiac Surgery, Madras Medical Mission, Chennai, India; ^16^ Department of Cardiac Anaesthesiology, EPIC Hospital, Ahmedabad, India

**Keywords:** aortic valve, mitral valve, structural valve deterioration, surgical valve replacement, pericardial bioprosthesis

## Abstract

**Background:**

The Dafodil™-1 trial was designed to evaluate the clinical safety and performance of Dafodil™ pericardial bioprosthesis for replacing diseased native or prosthetic aortic or mitral valves in patients with advanced valvular heart disease (VHD).

**Methods:**

The Dafodil™-1 trial was a prospective, multicenter, first-in-human clinical trial. Patients were enrolled if they had advanced VHD requiring aortic valve replacement (AVR) or mitral valve replacement (MVR) with or without concomitant valve surgery and having surgical risk scores <4%. Major adverse cardiac events (MACE), including all-cause death, myocardial infarction (MI), and stroke; and hemodynamics were analyzed.

**Results:**

A total of 136 patients (aortic: 67 and mitral: 69) were enrolled in the trial (with mean age—AVR group: 60.2 ± 8.3 years and MVR group: 49.7 ± 14.4 years). A total of 134 patients (aortic: 66 and mitral: 68) completed the 3-year follow-up (total 300 per 100 patient-years of follow-up). The AVR group demonstrated a significant reduction in the mean pressure gradients from 51.2 ± 24.1 mmHg at baseline to 11.1 ± 6.0 mmHg at the 3-year follow-up (*p* < 0.0001). The mean effective orifice area (EOA) improved from baseline (0.9 ± 0.6 cm^2^) to 3-year follow-up (1.8 ± 0.4 cm^2^) (*p* < 0.0001). In the MVR group, the mean indexed EOA (iEOA) increased significantly from baseline (0.7 ± 0.4 cm^2^/m^2^) to 3-year follow-up (1.1 ± 0.4 cm^2^/m^2^) (*p* < 0.001). There was significant improvement in New York Heart Association functional class and mean SF-12 scores in both groups. At 3-year follow-up, the MACE incidence was 2.3% per 100 patient-years (1.3% strokes per 100 patient-years and 1.3% deaths per 100 patient-years) for AVR group and 4.7% per 100 patient-years (0.6% strokes per 100 patient-years and 4.0% deaths per 100 patient-years) for MVR group. No cases of MI, structural valve deterioration and prosthetic valve endocarditis were reported. The AVR and MVR groups achieved 89.6% and 79.7% MACE-free survival, respectively at 3-year follow-up.

**Conclusions:**

The Dafodil™-1 trial demonstrated satisfactory outcomes of clinical safety, hemodynamic performance, and quality-of-life metrics. Additionally, no incidence of structural valve deterioration and very low rates of valve thrombosis during the 3-year follow-up period of Dafodil™-1 first-in-human trial indicated acceptable valve durability up to three years and similar outcomes are warranted for longer follow-ups as a primary goal.

**Clinical Trial Registration Number:**

https://www.ctri.nic.in/Clinicaltrials/showallp.php?mid1=18377&EncHid=&userName=CTRI/2017/07/009008, CTRI/2017/07/009008.

## 1 Introduction

Surgical valve replacement (SVR) remains the mainstay of treatment for valvular heart disease (VHD) affecting any of the four valves if they are not suitable for repair (1, 2). Over six decades, mechanical or tissue valve prostheses have been used for SVR, and the advantages and disadvantages of both are well documented (1, 3, 4). Newer surgical bioprosthetic heart valves (BHVs) have demonstrated a marked improvement in durability (2, 3, 5). The new-generation BHVs are made from corrosion-resistant frame material, subjected to anti-calcification/mineralization treatments, and have glutaraldehyde-fixed independent tissue leaflets (6). While there are substantial clinical data on the durability and hemodynamic performance of aortic tissue valves, their implantation in the mitral position remains less researched. However, there is comparatively limited recent evidence about the advantages of pericardial valve over a porcine prosthesis for mitral valve replacement (7).

An epidemiological report by the Centers for Disease Control and Prevention highlighted that of all deaths due to VHD in 2017, 61% and 15% deaths were attributed to aortic and mitral valve disease, respectively (8). Further, >90% patients with advanced VHD were aged <60 years in a study conducted in northern India (9).

SVR has become preferable over annuloplasty and/or valvuloplasty in patients aged <60 years with aortic and/or mitral valve disease, due to the improvements in BHVs (1, 10), and tissue valves composed of bovine pericardium or porcine tissue are preferred over mechanical valves (1, 2).

**Figure 1:**
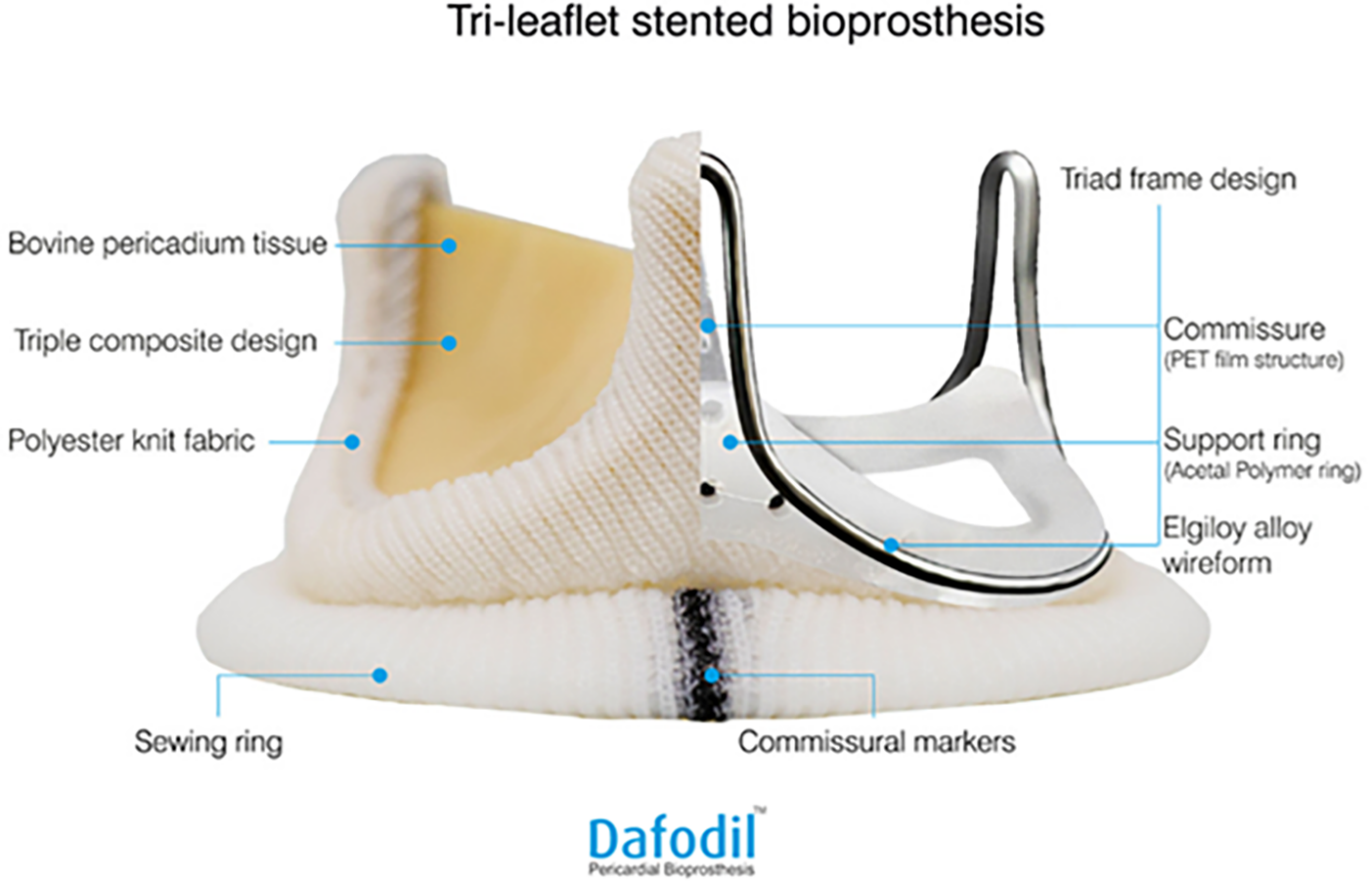
Device description of Dafodil™ pericardial bioprosthesis. Reprinted with permission from Meril Life Sciences Pvt Ltd. Adapted from Hiremath et al. (12), licensed CC BY 4.0.

BHVs can be stented or stent-less, and made of porcine tissue or bovine pericardium. Stented valves provide a good structural frame that prevents damage during implantation and ensures long-term maintenance of the valve geometry (11). The Dafodil™ pericardial bioprosthesis (Meril Life Sciences Pvt Ltd., India) is a tri-leaflet bovine pericardial valve, which has been indigenously developed in India. It has a stented design with three independent laser-cut leaflets, providing better leaflet coaptation and tenacious protection against leaflet calcification (Figure 1). The Dafodil^TM^ valve is designed for supra-annular placement, which aims to provide a larger effective orifice area (EOA), as the stent and the sewing ring are positioned on top of the native annulus. The valve has a sewing ring with commissural markers that aim to facilitate its attachment with appropriate orientation, especially useful in mitral valve replacement (MVR). The three leaflets are treated with proprietary anti-calcification technology (AntiCa^+^), with an aim to prevent calcification and valve degeneration. The Dafodil™ valve offers a comprehensive range of sizes for both aortic and mitral valves, tailored to accommodate the diverse anatomical requirements of patients undergoing valve replacement procedures. Aortic valve sizes in the Dafodil™ range from 19 mm to 27 mm, with options such as 19 mm, 21 mm, 23 mm, 25 mm, and 27 mm available. Similarly, mitral valve sizes in the Dafodil™ typically include 23 mm, 25 mm, 27 mm, 29 mm, and 31 mm, providing a wide selection to match individual patient needs. These precise size variations enable cardiac surgeons to choose the most appropriate valve size for each patient, ensuring optimal post-surgical functionality and compatibility.

The first-in-human trial evaluated the safety and clinical efficacy of Dafodil™ pericardial bioprosthesis. In the previous publication data of first 60 patients at 1-year has been presented (12). The current investigation reports the 3-year outcomes of 136 patients enrolled in the Dafodil™-1 trial.

## 2 Patients and methods

### 2.1 Ethics statement

All patients signed the informed consent form prior to their enrollment in the trial. The independent ethics committees of each participating institution approved the trial protocol and supervised the ethical conduct of the trial at each clinical site.

### 2.2 Study design

The Dafodil™-1 trial is a first-in-human, prospective, multicenter study enrolled patients across 19 centers in India between July 2017 and July 2019 to investigate the clinical safety and performance of the Dafodil™ pericardial Bioprosthesis.

The inclusion criteria were (i) age ≥18 years, low Society for Thoracic Surgeons (STS) risk scores (<4%), and indication for SVR following echocardiographic evaluation and assessment of medical history, coagulation profile (partial prothrombin time and international normalized ratio); (ii) planned aortic valve replacement (AVR)/MVR with or without coronary artery bypass grafting (CABG) or other valvular surgery; (iii) significant aortic/mitral stenosis or regurgitation, or combined aortic/mitral valve disease (stenosis and regurgitation). The main exclusion criteria were (i) active endocarditis/myocarditis or a recent 3-month history of endocarditis/myocarditis, stroke, cerebrovascular accident within last 6-months, or myocardial infarction (MI) within last 30-days, (ii) presence of non-cardiac disease limiting life expectancy to <5 years; (iii) concomitant end-stage renal disease requiring dialysis at screening; (iv) renal insufficiency based on serum creatinine levels, (v) hypertrophic obstructive cardiomyopathy; (vi) left ventricular ejection fraction (LVEF) ≤20% prior to planned valve surgery; (vii) presence of intra-cardiac mass, thrombus, or vegetation; (viii) presence of stroke, cerebrovascular accident (CVA) or transient ischemic attack (TIA) within 6 months and acute MI within 30 days prior to planned valve surgery; (ix) history of substance (drug or alcohol) abuse within the last 5 years prior to screening date; (x) concomitant left ventricular assist device (LVAD) placement; (xi) hemodynamic or respiratory instability requiring inotropic support, mechanical circulatory support, or mechanical ventilation within 30 days prior to planned valve surgery; (xii) documented leukopenia (WBC <3.5 × 10^3^ /μl), acute anemia (Hgb <10.0 gm/dl or 6 mmol/L) or thrombocytopenia (platelet count <50 × 10^3^ /μl) accompanied by history of bleeding diathesis and coagulopathy; (xiii) prior organ transplant or is currently an organ transplant candidate; (xiv) diagnosed with abnormal calcium metabolism and hyperparathyroidism; (xv) a known hypersensitivity or contraindication to antiplatelet drugs, anticoagulant drugs, polyethylene terephthalate, elgiloy and polyester.

### 2.3 Patient evaluation

At baseline, patients were evaluated for demographics, vital parameters, and comorbidities. Before surgery, the following parameters were analyzed on echocardiography: affected valve area, cardiac output index, LVEF, aortic annulus, mean and peak valvular pressure gradients, cardiac output, left ventricular systolic function, dimensions and volumes of the left ventricle, pressure half time, regurgitation grading, estimated right ventricular systolic pressure, pulmonary arterial systolic pressure, and mitral leaflet characteristics. All echocardiograms were independently analyzed by an echocardiographic core lab (CBCC Global Research LLP, India). Following the procedure, a structured postoperative follow-up regimen was recommended, comprising in-clinic follow-ups at 1-month, 6-month, 1-year, 2-year and 3-year. These intervals were designed to assess patient outcomes, monitor for any complications, and optimize long-term health. Further, the study has planned follow-ups at 4- and 5-year. The indexed effective orifice area (iEOA) calculated using Bernoulli's continuity equation is considered for the assessment as per the recommendations of the American Society of Echocardiography (ASE) (13). The required valve size to be implanted was measured by using a sizer provided by the manufacturer. The SVRs were performed according to the best practices at every institution, with adherence to the concurrent clinical practice recommendations (14). The decision on the prescription of antiplatelet therapy after implantation was left to the surgeons' discretion based on individual patient factors and clinical judgment.

### 2.4 Outcome measures/endpoints

The primary safety endpoints were (i) major adverse cardiovascular events (MACE), defined as a composite of all-cause mortality, MI, and stroke (ii) cardiovascular mortality, defined as all valve-related deaths due to structural or non-structural valve dysfunction, or other device-related and procedure-related deaths, including those related to a complication of the procedure or treatment for a complication of the procedure or death due to proximate cardiac cause (e.g., MI, cardiac tamponade, worsening heart failure). Secondary safety endpoints included stroke, major bleeding leading to rehospitalization, acute kidney injury (AKI) based on serum creatinine levels, valve thrombosis, and structural valve deterioration (SVD) [change in the function of a heart valve substitute resulting from an intrinsic abnormality that causes stenosis or regurgitation (including intrinsic changes such as wear, fatigue failure, stress fracture, occluder escape, suture line disruption of components)], valve-endocarditis, major paravalvular leakage, conduction disturbances and arrhythmias, nonstructural valve dysfunction (damage or dysfunction of the valve, explant, or hemolysis), early (all-cause deaths <30 days) and late mortality (all-cause deaths >30 days), and valve-related reoperation. The primary and secondary safety endpoints were evaluated at post-procedure (discharge), 1 month, 6 months, 1 year, 2 years and 3 years. For the surgical procedures, the approaches followed were median sternotomy, mini-sternotomy, or mini-thoracotomy as per the surgeon's discretion. The New York Heart Association (NYHA) functional assessment and Short Form (SF)-12v1 questionnaire were used to assess the quality of life (QoL) of the patients (15).

### 2.5 Statistical Analysis

Statistical data analysis was performed on the intention-to-treat population. Regarding the sample size, a minimum number of patients necessary for clinical evaluation were enrolled. The minimum sample size required to conduct the study was calculated based on the non-probability sampling method. The trial was designed to enroll an estimated minimum of 120 patients (60 in AVR and 60 in MVR) undergoing SVR with Dafodil™ pericardial bioprosthesis, including drop-out, on the basis of a non-probability sampling method to evaluate the clinical safety and performance of the Dafodil™ Pericardial Bioprosthesis. Continuous data are presented as mean with standard deviation (mean ± SD) and categorical data presented as numbers with percentages. The events related to patient survival were analyzed with Kaplan-Meier survival curves and the cumulative frequency of events were summarized as %events per 100 patient-years. All data were analyzed using the Statistical Package for the Social Sciences (SPSS) v.22.0, (IBM, Armonk, New York, USA).

## 3 Results

**Figure 2:**
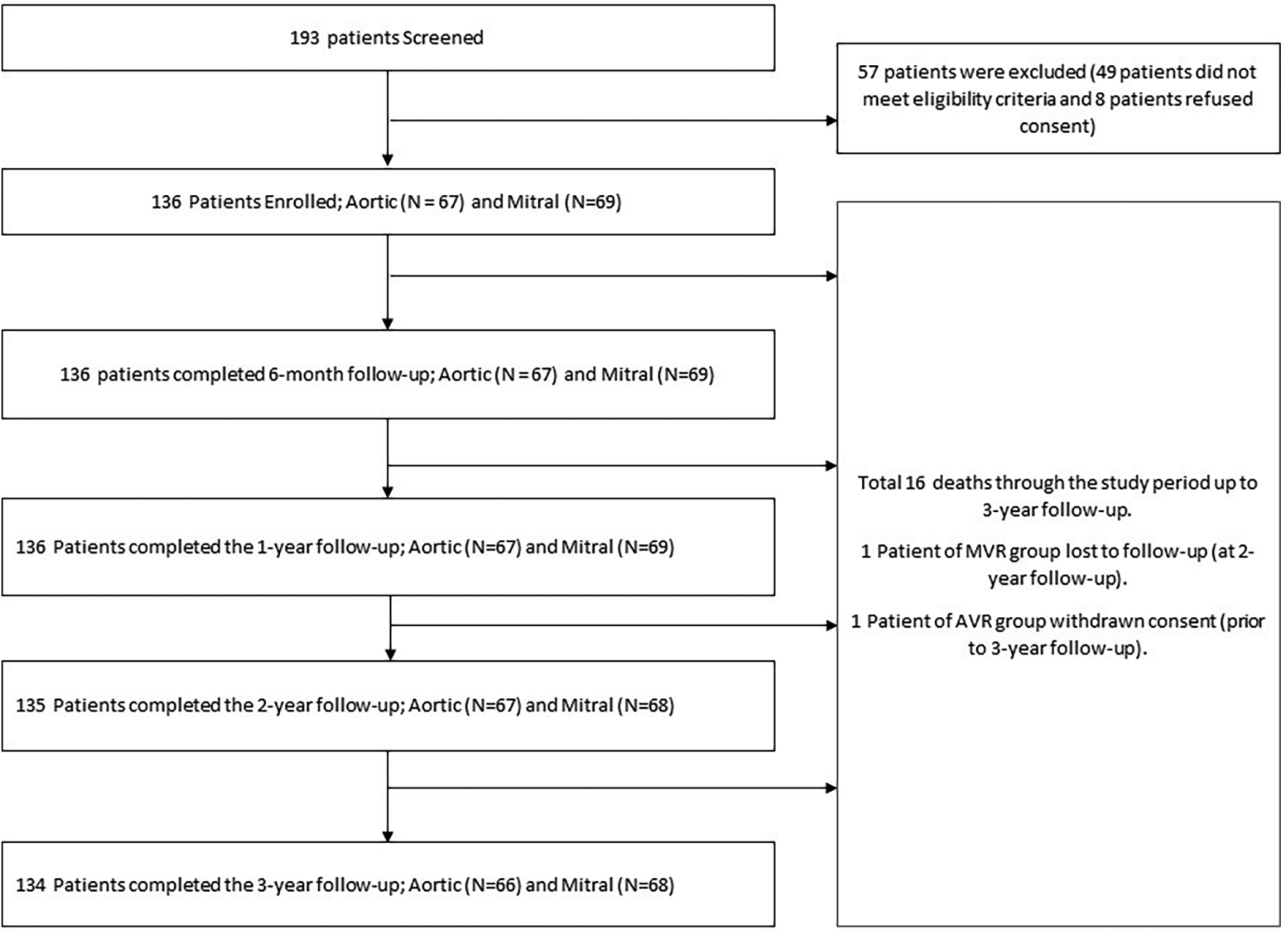
CONSORT flow diagram.

A total of 193 patients were screened, out of which 49 patients did not meet the eligibility criteria, 8 patients refused informed consent, hence a total of 136 patients were enrolled (aortic group: 67 and mitral group: 69). One patient in the AVR group withdrew consent prior to the 3-year follow-up and one patient from the MVR group was lost to follow-up prior to the 2-year follow-up. At the time of reporting, 134 patients had completed the 3-year follow-up (300 per 100 patient-years) including 66 and 68 patients in the AVR and MVR groups, respectively. The patient flow diagram according to the CONSORT (Consolidated Standards of Reporting Trials) guidelines is presented in Figure 2.

### 3.1 Baseline characteristics

The baseline demographic data of patients are summarized in Table 1.

**Table 1:** Baseline characteristics.

Variables	AVR group; n/*N* = 67/136	MVR group; n/*N* = 69/136	*p*-value
Age, years	60.2 ± 8.3	49.7 ± 14.4	<0.0001
Gender, *n* (%)			
Male	44 (65.7)	28 (40.6)	0.0058
Female	23 (34.3)	41 (59.4)	
Body mass index, kg/m^2^	24.2 ± 4.8	21.6 ± 5.0	0.0030
Body surface area, m^2^	1.6 ± 0.2	1.5 ± 0.3	0.0157
Cardiac status, *n* (%)			
Stable angina	7 (10.5)	4 (5.8)	0.4965
Unstable angina	0	1 (1.5)	1.0
Asymptomatic	60 (89.6)	64 (92.8)	0.7221
Cardiac rhythm, *n* (%)	*n* = 54	*n* = 44	
Sinus rhythm	1 (1.9)	32 (72.7)	<0.0001
Atrial fibrillation	52 (96.3)	11 (25.0)	
Atrial flutter	1 (1.9)	1 (2.3)	
Medical history, %			
Diabetes mellitus	13.4	1.5	0.0083
Hypertension	28.4	10.1	0.0130
Myocardial infarction	6.0	0	0.0562
Family history of CAD	6.0	0	0.0562
Cerebrovascular events	3.0	2.9	1.0
Congestive heart failure	3.0	1.5	0.6167
Heart rate, bpm, mean ± SD	77.1 ± 13.0	79.5 ± 13.4	0.9753
Diastolic blood pressure, mmHg, mean ± SD	73.4 ± 9.1	76.3 ± 13.9	0.0585
Systolic blood pressure, mmHg, mean ± SD	123.4 ± 16.6	120.8 ± 15.6	0.7252
Haemodynamic variables, mean ± SD			
Peak Pressure gradient (mmHg)	80.4 ± 4.0	16.6 ± 8.3	NA
Mean pressure gradient (mmHg)	51.2 ± 24.1	8.8 ± 5.0	NA
EOA (cm^2^)	0.9 ± 0.6	1.1 ± 0.6	NA
iEOA (cm^2^/m^2^)	0.5 ± 0.3	0.7 ± 0.4	NA
LVEF, %	54.8 ± 12.8	NA	NA
NYHA functional class, *n* (%)			
Class-I	3 (4.5)	2 (2.9)	0.045
Class-II	10 (14.9)	5 (7.2)	
Class-III	54 (80.6)	59 (85.5)	
Class-IV	0	3 (4.3)	

AVR, aortic valve replacement; CAD, coronary artery disease; MVR, mitral valve replacement; EOA, effective orifice area; iEOA, indexed EOA; LVEF, left ventricular ejection fraction; NYHA, New York Heart Association. Data are presented as mean ± SD or *n* (%).

#### 3.1.1 AVR group

**Figure 3:**
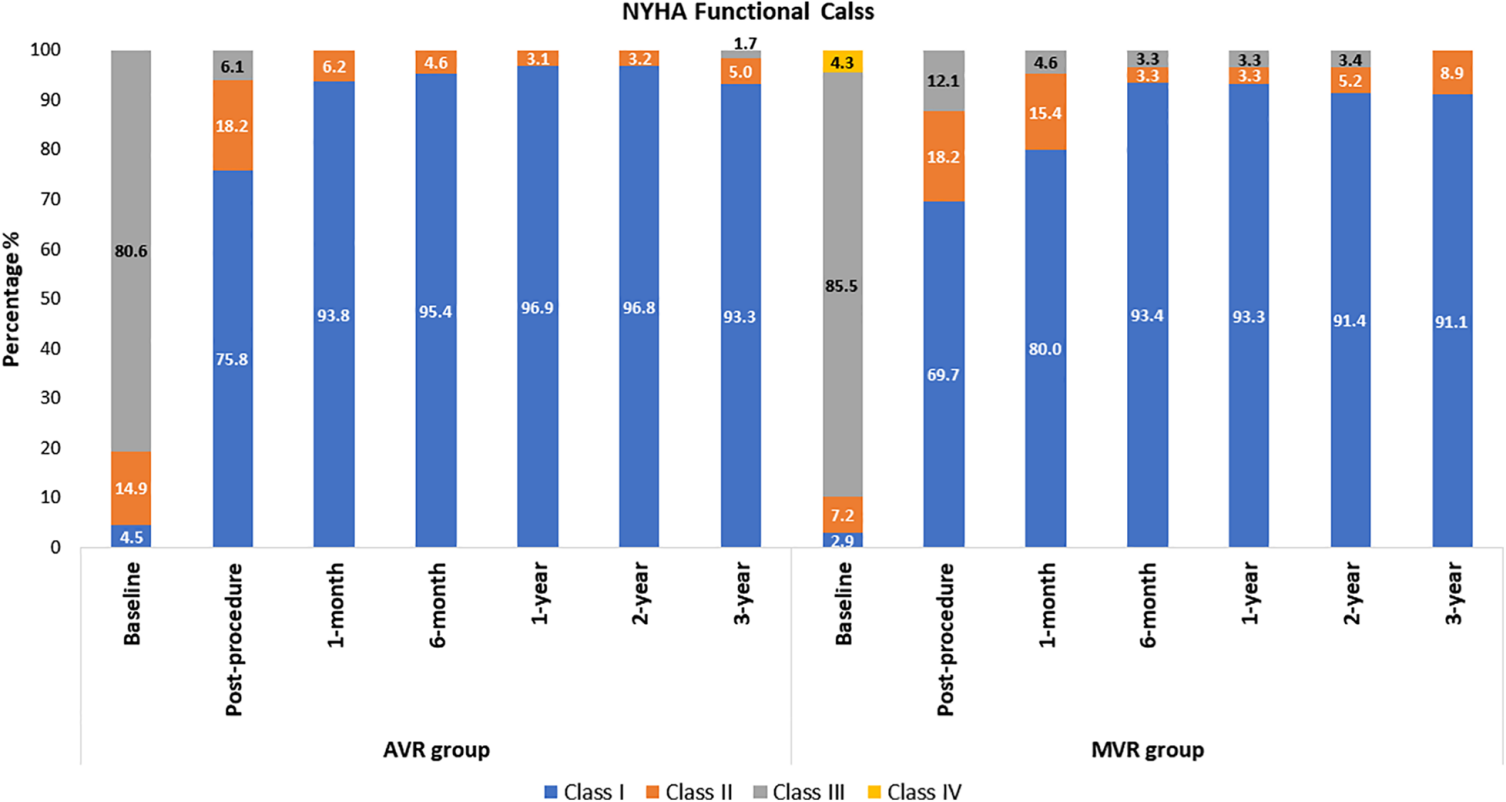
NYHA functional class: AVR and MVR groups.

The mean age of AVR patients was 60.2 ± 8.3 years, and 65.7% were male. The AVR group had low surgical risk scores (Society of Thoracic Surgeons predicted risk of mortality (STS-PROM); the mean score was 1.3 ± 0.7%. Patients’ medical history and cardiac status are shown in Table 1. At baseline, only 4.5% of patients were in the NYHA class I, and this improved to 75.8% at post-procedure and 93.3% at 3-year (Figure 3).

The baseline echocardiography evaluation data are shown in Table 2. The mean EOA at baseline was 0.9 ± 0.6 cm^2^ and the mean aortic annulus diameter was 23.7 ± 2.9 mm, indicating the prevalence of small aortic annulus (SAA) in the study population.

**Table 2:** Echocardiographic analysis data.

Parameters	Baseline	Discharge	1-month	6-month	1-year	3-year
AVR Group						
BSA, m^2^	1.6 ± 0.2	1.6 ± 0.2	1.6 ± 0.2	1.6 ± 0.2	1.6 ± 0.2	1.7 ± 0.3
Heart rate, bpm	75.9 ± 14.8	79.3 ± 11.2	74.5 ± 11.4	74.9 ± 10.3	72.2 ± 11.8	70.5 ± 10.2
VTI (LVOT), cm	25.0 ± 10.4	19.4 ± 6.2	21.5 ± 5.8	24.3 ± 6.3	22.5 ± 4.5	26.0 ± 5.1
VTI (AV), cm	99.1 ± 30.7	38.8 ± 14.3	40.9 ± 9.1	46.5 ± 9.7	48.3 ± 11.5	46.3 ± 11.2
LVOT diameter, mm	2.2 ± 0.3	2.0 ± 0.2	2.0 ± 0.2	2.0 ± 0.2	2.1 ± 0.2	2.0 ± 0.2
Peak Pressure Gradient (mmHg)	80.4 ± 34.0	25.5 ± 14.3	23.1 ± 6.7	23.1 ± 7.1	24.6 ± 9.7	21.3 ± 12.2
Mean Pressure Gradient (mmHg)	51.2 ± 24.1	13.9 ± 7.6	12.4 ± 3.8	12.5 ± 4.5	13.8 ± 6.3	11.1 ± 6.0
EOA (cm^2^)	0.9 ± 0.6	1.5 ± 0.5	1.6 ± 0.4	1.7 ± 0.4	1.7 ± 0.4	1.8 ± 0.4
iEOA (cm^2^/m^2^)	0.5 ± 0.3	0.9 ± 0.3	1.0 ± 0.2	1.1 ± 0.3	1.0 ± 0.3	1.1 ± 0.3
Stroke volume, ml	79.2 ± 26.9	53.2 ± 19.6	60.7 ± 17.0	75.7 ± 20.9	75.6 ± 22.1	74.7 ± 26.4
Stroke volume index, ml/min	49.7 ± 19.2	32.7 ± 11.8	37.8 ± 10.4	45.6 ± 14.6	46.8 ± 12.6	44.7 ± 17.4
Cardiac output, lit/min	5.7 ± 1.8	4.3 ± 1.8	4.5 ± 1.5	5.7 ± 1.5	5.5 ± 1.9	5.4 ± 1.7
Cardiac index, lit m^−2^ min^−1^	3.5 ± 1.2	2.6 ± 1.1	2.9 ± 0.9	3.5 ± 1.1	3.4 ± 1.1	3.3 ± 1.2
LVEF, %	54.8 ± 12.8	55.5 ± 10.3	58.7 ± 7.3	60.2 ± 6.6	59.9 ± 8.0	57.6 ± 8.0
LVESV, ml	59.7 ± 37.0	42.7 ± 28.6	38.6 ± 19.2	35.7 ± 18.6	34.2 ± 14.7	41.0 ± 22.8
LVEDV, ml	126.2 ± 42.0	95.3 ± 38.3	93.3 ± 32.7	93.6 ± 27.7	90.2 ± 27.3	93.3 ± 35.9
Interventricular septum, mm	14.8 ± 3.4	13.8 ± 2.7	13.5 ± 2.5	13.3 ± 3.0	13.5 ± 3.2	13.3 ± 3.3
MVR Group						
Peak pressure gradient, mmHg	16.6 ± 8.3	10.2 ± 4.2	9.2 ± 3.8	9.6 ± 3.6	10.5 ± 5.9	10.9 ± 4.4
Mean pressure gradient, mmHg	8.8 ± 5.0	3.9 ± 1.6	3.7 ± 1.8	3.9 ± 1.5	4.4 ± 2.8	4.4 ± 1.7
EOA, cm^2^	1.1 ± 0.6	1.8 ± 0.5	1.9 ± 0.5	1.6 ± 0.4	1.7 ± 0.7	1.6 ± 0.5
iEOA, cm^2^/m^2^	0.7 ± 0.4	1.3 ± 0.4	1.2 ± 0.4	1.1 ± 0.3	1.1 ± 0.5	1.1 ± 0.4
VTI (MV), cm	66.2 ± 31.3	34.2 ± 8.2	34.4 ± 9.5	39.9 ± 10.3	39.9 ± 11.2	42.5 ± 12.3
Cardiac output, lit/min	4.5 ± 1.7	4.3 ± 1.4	4.6 ± 1.4	4.3 ± 1.5	4.6 ± 1.7	3.9 ± 1.1

AV, aortic valve; LVEDV, left ventricular end-diastolic volume; LVEF, left ventricular ejection fraction; LVESV, left ventricular end-systolic volumes; LVIDd, left ventricular internal dimensions at end-diastole; LVIDs, left ventricular internal dimensions at end-systole; LVOT, left ventricular outflow tract; VTI, velocity time integral.

Data are presented as mean ± SD.

#### 3.1.2 MVR group

The MVR patients had a mean age of 49.7 ± 14.4 years and 40.6% were male. Their mean STS score was 1.7 ± 1.3%. Patients' medical history and cardiac status are shown in Table 1. The baseline echocardiography evaluation data is shown in Table 2. At baseline, only 2.9% of patients had NYHA class I, and improved to 69.7% at post-procedure and 91.1% at 3-year and none of the patients belonged to Class III and IV (Figure 3). The mean affected valve area was 1.1 ± 0.6 cm^2^; 55.9% of patients had a calcified mitral annulus, whereas 44.1% had non-calcified mitral annulus. The average mitral annulus diameter was 33.9 ± 5.1 mm.

### 3.2 Procedural characteristics

#### 3.2.1 AVR group

A total of 67 patients underwent AVR. Coronary artery bypass grafting (CABG) was the most frequent concomitant procedure (13.4%) in the AVR group; other concomitant surgeries performed were ascending aorta replacement (1.5%), root enlargement (6.0%), mitral valve repair (7.5%), and dual valve replacement (1.5%). 71.6% of patients underwent isolated AVR. The majority of patients underwent full sternotomy (97.0%), while mini-sternotomy and mini-thoracotomy were each performed in 1.5% of patients. The distribution of valve sizes was as follows: 19-, 21-, 23-, and 25-mm valves in 43.3%, 38.8%, 13.4%, and 4.5% of patients, respectively. The required valve size to be implanted was measured by using a sizer provided by the manufacturer. The implantation technique used was interrupted in majority of the patients (93.1%). Pledgeted suturing was used in 79.3% of the patients. The mean cardiopulmonary bypass (CPB) time in the AVR group was 134.33 ± 54.93 min; mean aortic cross-clamping time: 95.04 ± 39.48 min.

#### 3.2.2 MVR group

In the MVR group, tricuspid valve repair (30.4%) was the most frequently performed concomitant surgery followed by CABG (8.7%), and dual valve replacement (1.5%). 60.9% of patients underwent isolated MVR. Most patients (97.1%) underwent full sternotomy while the rest (2.9%) underwent lower mini-sternotomy. The mean CPB time and aortic cross-clamp time was 118.1 ± 40.1 min and 82.2 ± 23.2 min, respectively. The 27-mm valve size was the most frequently used (31.9%), followed by 31-mm (24.6%), 29-mm (21.7%), 25-mm (20.3%), and 23-mm (1.5%) sizes. The required valve size to be implanted was measured by using a sizer provided by the manufacturer. The implantation technique used was interrupted in majority of the patients (87.7%). Pledgeted suturing was used in 72.3% of the patients.

The mean length of hospital stay was 7.6 ± 3.4 days in AVR group and 8.8 ± 10.4 days in MVR group.

### 3.3 Outcomes at 3-year follow-up

Table 3 represents the primary and secondary outcomes data through 3-year follow-up.

**Table 3:** Clinical outcomes of Dafodil™-1 trial.

Events, *n* (%)	Early outcomes						Late outcomes			
	Post-Procedure (Discharge) (*n*=136)		1-Month FU (*n*=136)		6-Month FU (*n*=136)		1-year FU (*n*=136)^a^		3-year FU^b^ (*n*=134) Cumulative events (% events per 100 patient-years)	
	Aortic (*n*=67)	Mitral (*n*=69)	Aortic (*n*=67)	Mitral (*n*=69)	Aortic (*n*=67)	Mitral (*n*=69)	Aortic (*n*=67)	Mitral (*n*=69)	Aortic (*n*=66)	Mitral (*n*=68)
All-cause mortality	1 (1.5)	3^c^^,^^d^ (4.3)	2 (3.0)	4 (5.8)	2 (3.0)	7 (10.1)	2 (3.0)	9 (13.0)	4 (1.3)	12^g^ (4.0)
Cardiovascular death	0	2 (3.0)	1 (1.5)	3 (4.3)	1 (1.5)	4 (5.8)	1 (1.5)	6 (8.7)	2 (0.7)	9 (3.0)
MI	0	0	0	0	0	0	0	0	0	0
Stroke	0	0	1 (1.5)	0	1 (1.5)	1 (1.4)	3^e^ (4.5)	2 (2.9)	4^f^ (1.3)	2 (0.6)
MACE	1 (1.5)	3 (4.3)	3 (4.5)	4 (5.8)	3 (4.5)	8 (11.6)	5 (7.5)	11 (15.9)	7 (2.3)	14 (4.7)
All bleeding (major/minor)	0	1^d^ (1.4)	0	1 (1.4)	0	1 (1.4)	0	1 (1.4)	0	1 (0.3)
Acute kidney injury	0	1^d^ (1.4)	0	1 (1.4)	0	1 (1.4)	0	1 (1.4)	0	1 (0.3)
Valve thrombosis	0	1^c^ (1.4)	0	1 (1.4)	0	1 (1.4)	0	1 (1.4)	0	1 (0.3)
SVD	0	0	0	0	0	0	0	0	0	0
Repeat hospitalization	0	0	4 (6.0)	5 (7.2)	5 (7.5)	7 (10.1)	8 (11.9)	9 (13.0)	11 (3.7)	13 (4.3)
Conduction disturbances/PPI	1 (1.5)	0	2 (3.0)	0	2 (3.0)	0	2 (3.0)	0	2 (0.7)	0
Prosthetic valve endocarditis	0	0	0	0	0	0	0	0	0	0
Major paravalvular leak	0	0	0	0	0	0	0	0	0	1 (0.3)
Explant	0	1^c^ (1.4)	0	1 (1.4)	0	1 (1.4)	0	1 (1.4)	0	2^g^ (0.7)
Hemolysis	0	0	0	0	0	0	0	0	0	0
Valve related re-operation	0	1^c^ (1.4)	0	1 (1.4)	0	1 (1.4)	0	1 (1.4)	0	2^g^ (0.7)

SVD, Structural valve deterioration.

^a^ The clinical outcomes of all FU visits up to 1-year FU are given as *n* (%).

^b^ The event rates at the 3-year FU are reported as %events per total patient-years of follow-up (total follow-up = 300 per 100 patient-years).

^c^ One patient had valve thrombosis post-procedure and had to re-operate. The patient eventually died.

^d^ One patient had excessive bleeding on post-operative and succumbed to death due to acute kidney injury.

^e^ One patient suffered from brain stroke and eventually died.

^f^ One patient suffered from CV stroke.

^g^ One patient died due to multi-organ dysfunction who underwent redo surgery.

MACE is defined as a composite of all-cause mortality, MI, and stroke.

#### 3.3.1 AVR group

**Figure 4:**
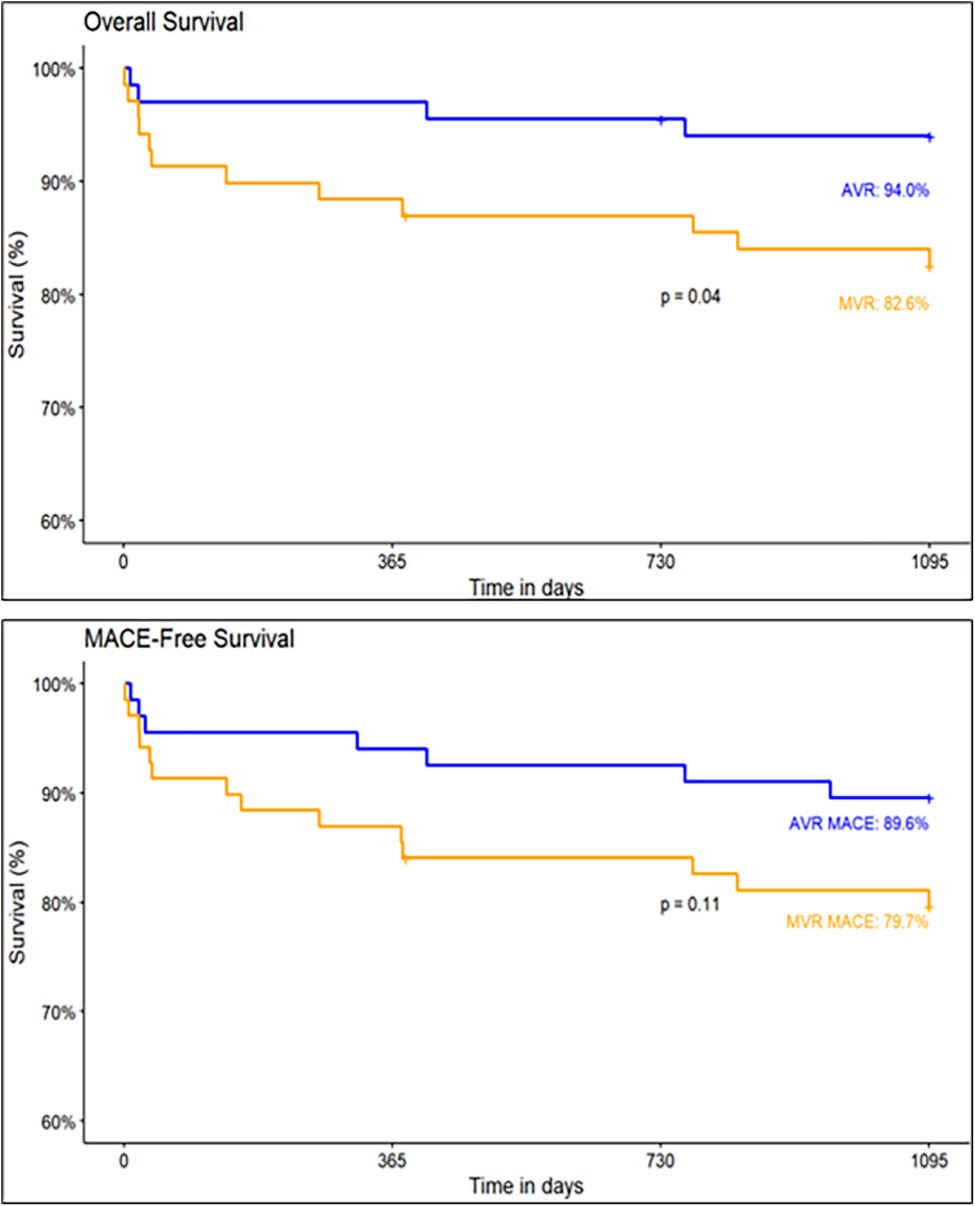
Kaplan–meier estimates of MACE-free and overall survival in AVR and MVR groups.

At discharge, the MACE and cardiovascular mortality rates were 1.5% and 0%, respectively. At 1-year follow-up, the MACE and cardiovascular mortality rates were 7.5% and 1.5%, respectively. The MACE rate at the 3-year follow-up was 2.3% per 100 patient-years. Including 1.3% stroke, 1.3% all-cause mortality events per 100 patient-years. No incidences of structural valve deterioration, MI, major bleeding, acute kidney injury, valve thrombosis, prosthetic valve endocarditis, and hemolysis were observed. The MACE-free survival for the AVR group was 89.6% and the overall survival was 94% (Figure 4).

#### 3.3.2 MVR group

At discharge, the MACE and cardiovascular mortality rates were 4.3% and 3.0%, respectively. At 1-year follow-up, the MACE and cardiovascular mortality rates were 15.9% and 8.7%, respectively. The MACE rate at the 3-year follow-up was 4.7% per 100 patient-years. Including 0.6% stroke and 4.0% all-cause mortality events per 100 patient-years. At the completion of 3-year, major and minor bleeding cases, acute kidney injury and valve thrombosis was 0.3% per 100 patient-years. No cases of MI, structural valve deterioration, conduction disturbances, prosthetic valve endocarditis, and hemolysis were observed. In the MVR group, the MACE-free survival was 79.7% and the overall survival was 82.6% (Figure 4).

In both the groups (AVR and MVR groups) there were no incidences of structural valve deterioration, MI, prosthetic valve endocarditis, and hemolysis.

The overall survival (94.0% vs. 82.6%, *p* = 0.04) and MACE-free survival (89.6% vs. 79.7%, *p* = 0.11) rates at 3-year follow-up are higher in AVR group than in MVR group (Figure 4).

Early mortalities [3.0% in AVR group and 5.8% in MVR group] were reported in high-risk comorbid patients. The causes of early mortality include myocardial failure, cardiopulmonary arrest, multi-organ failure, mesenteric ischemia, ventricular arrhythmia and congestive cardiac failure. Among all these deaths, one death was device-related, while others were not related to the device.

All other cases of serious adverse events were resolved successfully, including one case of stroke in a patient who underwent MVR with Dafodil™. The patient developed a stroke five months after surgery, and his clinical laboratory tests revealed elevated levels of homocysteine (28.8 μmol/L), HbA1c (7.1%), and erythrocyte sedimentation rate (28%). A brain MRI revealed a left acute mid-cerebral artery infarct in the peri-sylvian area. An echocardiogram showed normal hemodynamics of the bioprosthetic valve. Consequently, he received medical therapy, with periodic monitoring of coagulation profile. No signs of implantation surgery or device-related stroke were reported after a thorough investigation and the patient has remained stable.

At the 3-year follow-up, one AVR patient was diagnosed with an ascending aortic aneurysm. An echocardiographic evaluation showed normal function of bioprosthetic valve *in situ* with normal biventricular function (aortic valve peak gradient: 15 mmHg, mean gradient: 9 mmHg). The patient underwent surgical repair of the large aneurysm and further received antibiotics for methicillin-resistant Staphylococcus aureus (MRSA) infection. After a repeat rehospitalization event, he was discharged home in a hemodynamically stable condition.

### 3.4 Hemodynamic outcomes

#### 3.4.1 AVR group

**Figure 5:**
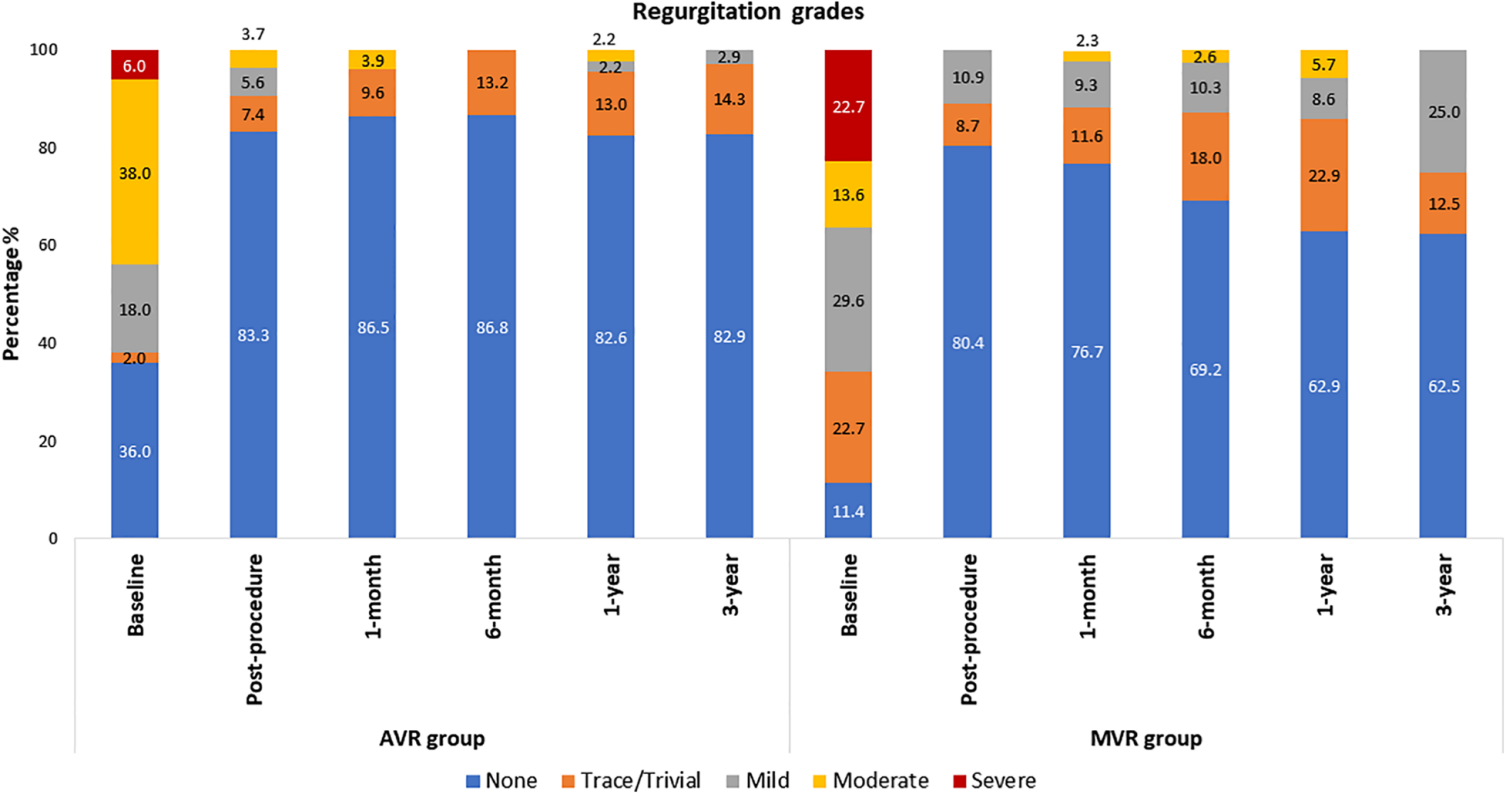
Regurgitation grades: AVR and MVR groups.

The echocardiographic outcomes are shown in Table 2. After valve implantation, post-procedure, none of the patients were reported with severe AR. 7.4% (*n* = 4; transvalvular − 4) and 5.6% (*n* = 3; transvalvular − 2 and paravalvular − 1) of the patients had trace/trivial and mild AR, respectively. Only 3.7% (*n* = 2; transvalvular − 1 and paravalvular − 1) of patients had moderate AR post-procedure. At 3-year follow-up, none of the patients were reported with moderate or severe AR, while 14.3% and 2.9% had trace and mild AR, respectively (Figure 5). There was a marked improvement observed in mean pressure gradient (Baseline: 51.2 ± 24.1 mmHg; 3-year: 11.1 ± 6.0 mmHg; *p* < 0.0001) and EOA (Baseline: 0.9 ± 0.6 cm^2^; 3-year: 1.8 ± 0.4 cm^2^; *p* < 0.0001) at 3-year follow-up period with no worsening of AR (Table 2). As shown in Table 2, there were a significant (*p* < 0.001) improvement in the iEOA from baseline (0.5 ± 0.3 cm^2^/m^2^) to 3-year follow-up (1.1 ± 0.3 cm^2^/m^2^).

#### 3.4.2 MVR group

Table 2 shows the echocardiographic results of the MVR group. After valve implantation with the Dafodil™ pericardial bioprosthesis, no mitral regurgitation (MR) was observed in 80.4% of patients at discharge while none of the patients were reported with moderate or severe MR. However, 25% and 12.5% of patients had mild and trivial MR at the 3-year follow-up, respectively, while 62.5% of patients showed absence of MR and none of the patients had moderate/severe MR (Figure 5). The MVR group showed satisfactory hemodynamic improvements with the reduction in mean pressure gradient (8.8 ± 5.0 mmHg at baseline to 4.4 ± 1.7 mmHg at 3-year) and improvement in EOA (1.1 ± 0.6 cm^2^ at baseline to 1.6 ± 0.5 cm^2^ at 3-year; *p* < 0.001) (Table 2).

### 3.5 Quality of life

#### 3.5.1 AVR group

In the SF-12 score assessment, the physical (PCS) and mental component summary (MCS) scores showed significant improvements from baseline to the 3-year follow-up (baseline: PCS = 33.8 ± 7.4, MCS: 43.4 ± 9.6; 3-year: PCS: 49.0 ± 7.0, MCS: 54.9 ± 7.8; *p* < 0.001) (Supplementary Table S1).

#### 3.5.2 MVR group

The PCS and MCS scores in the SF-12 score assessment improved significantly from baseline to the 3-year follow-up (baseline: PCS: 32.1 ± 6.4, MCS: 43.5 ± 10.2; 3-year: PCS: 50.2 ± 7.4, MCS: 53.8 ± 7.4; *p* < 0.001) (Supplementary Table S1).

### 3.6 Patients with Small Aortic Annulus

The study included 55 patients requiring 19-mm and 21-mm sized valves. Statistically significant improvements were observed in the mean pressure gradient (15.4 ± 6.7 mmHg at discharge, 12.5 ± 6.3 mmHg at 3-year; *p* < 0.001) in patients who received 19-mm valves (Supplementary Table S2). Among patients who received 21 mm valves, the mean gradient reduced to 14.9 ± 9.0 mmHg at discharge, and was maintained up to 3-year follow-up (10.7 ± 6.5 mmHg) (Supplementary Table S2). The NYHA functional class also improved with 88% of patients who received the 19-mm Dafodil™ aortic valve in class I and 95.8% who received 21-mm Dafodil™ aortic valve were in class I at 3 years (Supplementary Table S3). Similarly, gradual improvements in the mean SF-12 scores were observed in the patients who received 19-mm or 21-mm Dafodil™ aortic valve (Supplementary Table S4). There were no cases of valve thrombosis, reoperation, explant, SVD, major or minor bleeding, new PPI and AKI, and minimal event rates of stroke (0.67 per 100 patient-years) and MACE (1.67 per 100 patient-years) over the 3-year follow-up (Supplementary Table S5).

## 4 Discussion

The results of the Dafodil™-1 trial showed favorable 3-year clinical safety and hemodynamic performance in patients who underwent AVR and MVR and received the Dafodil™ pericardial bioprosthesis. The MACE incidence was 2.3% per 100 patient-years (1.3% strokes per 100 patient-years and 1.3% deaths per 100 patient-years) for AVR group and 4.7% per 100 patient-years (0.6% strokes per 100 patient-years and 4.0% deaths per 100 patient-years) for MVR group. The Kaplan–Meier estimates of MACE-free survival for the AVR and MVR groups were 89.6% and 79.7% (*p* = 0.11). Previously, the 1-year results had reported favorable clinical safety and performance with the Dafodil™ prosthesis (12).

The hemodynamic performance of the AVR group in our study is comparable with that of the Inspiris Resilia™ aortic valve, which was investigated prospectively in a large multinational cohort by the COMMENCE trial investigators (16–18). Over the 5-year follow-up, similar hemodynamic improvements were observed for the overall study population; however, aortic root enlargement was concomitantly required for patients with SAA (16). The 4-year outcomes of the COMMENCE trial reported an all-cause mortality rate of 2.2% per patient-year, including 0.4% valve-related mortality per patient-year. In addition, the rate of thromboembolism events was 1.7% per patient-year (18). Our outcomes are comparable to these, thus demonstrating an acceptable device safety and performance. In the COMMENCE mitral cohort, the 5-year probability of event-free survival was 79.9% and all-cause mortality rate was 18.07%. There was one case of postoperative major paravalvular leak (PVL), which required reintervention with a PERIMOUNT Magna Mitral Ease valve and one case required reintervention (19).

In the PERIGON Pivotal trial, the estimated Kaplan-Meier cumulative probability of all late bleeding events and late major bleeding events at 3 years was 7.3% and 4.3%, respectively. The estimated Kaplan-Meier cumulative probability of all-deaths was 7.2% (95% confidence interval [CI]: 5.6–9.0) (20). In our study there were no major bleeding events. Moreover, 93.3% and 91.1% of patients in the AVR and MVR groups had NYHA class I status at the 3-year follow-up. Further, a significant impact on the health-related QoL was observed at the 3-year follow-up. In comparison, 75.3% patients were in class I in the PERIGON Pivotal trial at the 3-year follow-up following SAVR with the Avalus pericardial bioprosthesis (Medtronic Inc., Minneapolis, Minnesota, USA) [20]. This PERIGON trial also reported high transvalvular gradients in patients who received 19-mm (18.2 ± 6.1 mmHg at discharge, 18.3 ± 6.0 mmHg at 3-year) and 21-mm Avalus BHVs (15.2 ± 4.9 mmHg at discharge; 15.9 ± 5.5 mmHg at 3-year), while mild PVL was noted in 2.7% patients (21). Our outcomes are similar to these results.

Eichinger et al. conducted a study of 561 valve replacements (AVR: 461 and MVR: 100) (22) using the Mosaic™ bioprosthetic valve. They reported improvement in mean pressure gradients and EOA for AVR (12.8 ± 5.2 mmHg; 1.7 ± 0.5 cm^2^) and MVR (4.1 ± 1.4 mmHg; 1.7 ± 0.5 cm^2^) cohorts at the 5-year follow-up. Similar hemodynamic improvements were observed in the AVR and MVR groups in our study at the 3-year follow-up (AVR: 11.1 ± 6.0 mmHg, 1.8 ± 0.4 cm^2^ and MVR: 4.4 ± 1.7 mmHg and 1.6 ± 0.5 cm^2^).

We noted that the CPB and aortic cross-clamp times were significantly higher in the Dafodil™-1 trial for patients who underwent AVR with concomitant procedures (CPB: 163.8 ± 63.0 min; Aortic cross-clamp: 116.7 ± 53.1 min). We believe that these durations will reduce considerably with improved learning curve in future years. The CPB (113.2 ± 29.8 min) and aortic cross-clamp time (79.4 ± 22.8 min) was significantly lower in the MVR patients who underwent isolated MVR. Even among those who underwent concomitant procedures with MVR, the CPB (125.7 ± 52.1 min) and aortic cross-clamp duration (86.5 ± 23.5 min) was remarkably lower, which proves the ease of implantation of the Dafodil™ mitral valve. The Dafodil™ mitral valve has dedicated black suture lines that aid in implantation and orientation of the valve, which is further supported by the commissural markers on the sewing ring.

Overall, our data are comparable to the data reported in contemporary studies of pericardial bioprosthetic valves. With these mid-term follow-up results from the Dafodil™-1 trial, the suitability of Dafodil™ pericardial valve for the Asian population is well demonstrated. The AVR cohort of the Dafodil™-1 trial faired consistently with the data of recent Indian studies that analyzed the outcomes of valve replacements using various models of the Carpentier—Edwards porcine bioprosthesis and PERIMOUNT pericardial bioprosthesis in South Asian patients (5, 10). Talwar et al. reported 2.4% early and 0.7% late mortality in a cohort of 559 patients who underwent MVR, AVR, tricuspid, and double valve replacements (10).

Furthermore, contrary to the observation by Freitas-Ferraz et al. that stented valves may not be the best choice for treating patients with a small aortic annulus (23), the Dafodil™-1 trial showed significantly improved outcomes for the SAA patients enrolled, including good improvements in the hemodynamics, NYHA functional class, and SF-12 scores. Future long-term evaluation of this patient group would be critically useful to aid in the device selection in routine clinical practice.

The Dafodil™ pericardial bioprosthesis, Inspiris Resilia™ aortic valve, Avalus™ bioprosthesis, and Perimount Magna Ease™ are new-generation bioprosthetic valves used in surgical valve replacement. All these valves are made up of bovine pericardium. The Dafodil™ valve, crafted from bovine pericardial tissue, features a tri-leaflet design within an elgiloy alloy stent and includes a proprietary AntiCa^+^ anti-calcification treatment, addressing concerns such as calcification. The black sutures help reduce the commissural profile and ease the implantation. On the other hand, the Inspiris Resilia™ aortic valve incorporates a stented tri-leaflet design made from Resilia™ bovine pericardial tissue, and it utilizes EIP™ technology for anti-calcification. In contrast to the Dafodil™ bioprostheses, the Inspiris Resilia™ has a distinct valve tailored for mitral positioning, whereas the Dafodil™ valve can be employed for both AVR and MVR surgeries. The Avalus™ bioprosthesis is similar to that of other stented aortic bioprosthetic valves. It has Proprietary AOA™ anti-calcification technology which mitigates calcification and protects the tissue, but this valve is not suitable for use in the mitral valve positioning. Lastly, Perimount Magna Ease™ valve, tri-leaflet bioprosthesis, available for both aortic and mitral positions, has Thermafix anti-calcification technology and suture markers aid in valve orientation and suture placement.

With the increased use of Bioprosthesis for patients who require surgical valve replacement, SVD and the need for reintervention remains a concern, particularly in younger patients with long-life expectancy. Other than the above-mentioned studies, the long-term durability studies of the Epic, Mosaic, and Perimount Magna Ease bioprostheses are available. These studies primarily focus on the incidences of major bleeding events, SVD, and need for reoperation. The long-term outcomes of these studies, are given in Table 4. The Epic and Perimount Magna Ease bioprostheses have demonstrated acceptable results in terms of freedom from all-cause mortality and valve related complications, whereas the SVD rates of Mosaic bioprosthesis are comparable with the Perimount Magna ease bioprosthesis. Like the long-term studies of other devices, further follow-up of Dafodil™-1 first-in-human trial is ongoing.

**Table 4:** Literature overview for bioprostheses durability.

Valve	Follow-up	Reference	Survival rate/Freedom from all-cause mortality		Freedom from SVD		Freedom from reoperation/re-intervention		Freedom from bleeding events		Freedom from valve thrombosis		Freedom from Explant	
			AVR	MVR	AVR	MVR	AVR	MVR	AVR	MVR	AVR	MVR	AVR	MVR
Perimount Magna Ease valve	8 years	Tsui et al. 2022 (24)^a^	80.7% (74.9–86.4)	NA	90.1% (84.72–95.4)	NA	89.8% (85.1–94.6)	NA	85.1% (80.0–90.1)	NA	100% (100.0–100.0)	NA	94.8% (91.7–97.9)	NA
Mosaic valve	12 years	Jamieson et al. 2011 (25)^b^	55.8% ± 3.7%	43.9% ± 7.4%	93.3% ± 2.6%	NA	NA	NA	NA	NA	NA	NA	NA	NA
Mosaic valve	12 years	Yoshikawa et al. 2020 (26)^b^	59.9% ± 7.5%	NA	93.5% ± 2.9%	NA	86.4% ± 2.6%	NA	NA	NA	NA	NA	NA	NA
Mosaic Mitral valve	15 years	Celiento et al. 2016 (27)^b^	NA	37% ± 8%	NA	93% ± 5%	NA	91% ± 5%	NA	NA	NA	NA	NA	NA
Epic valve	5 years	Lehmann et al. 2007 (28)^b^	77.0% ± 4.1%	71.7% ± 4.5%	NA	NA	98.9% ± 0.7%	96.7% ± 1.9%	99.5% ± 0.2%	100%	NA	NA	NA	NA

NA, not available.

^a^ 95% Confidence interval.

^b^ Values are given in mean ± SD.

## 5 Limitations

We note that the sample size of the trial was a major limitation. The reasons for this can be linked to the low accessibility to quality healthcare among diseased populations in India, which is a middle-income country. The current study is on-going trial, which is not sufficiently powered to address structural valve deterioration over time. Hence, the long-term follow-up is warranted. The socioeconomic factors play a considerable role in the patients' decision-making regarding invasive treatment for VHD. However, the effects of these nonclinical factors are expected to reduce in the forthcoming years, as the access to affordable healthcare increases and the availability of this indigenously designed prosthetic valve grows. We acknowledge that the data of MVR patients may serve critically to evaluate the clinical need of a viable mitral valve prosthesis in the South Asian population, where aortic and mitral valve diseases are highly prevalent, even though there were limited number of patients.

## 6 Conclusion

This is a first-in-man study of Dafodil™ pericardial bioprosthesis for AVR and MVR, which demonstrated favorable 3-year outcomes and hemodynamic improvements, and significantly enhanced patients' quality of life. Further large-scale randomized trials with longer follow-ups are required to corroborate the findings of this first-in-man study.

## Data Availability

The raw data supporting the conclusions of this article will be made available by the corresponding author upon reasonable request.
