# Supplementary material for: Three-year outcomes of surgical valve replacement with Dafodil™ pericardial bioprosthesis: Dafodil™-1 trial
Source: Front Cardiovasc Med. 2024 May 30;11:1393762. doi: 10.3389/fcvm.2024.1393762 (PMC11171715; doi:10.3389/fcvm.2024.1393762)
Supplement: Supplementary file 1 [file Table1.docx]

Supplementary Material

1. **Supplementary Tables:**

**Supplementary Table S1. Improvements in the Mean SF-12 scores of AVR and MVR groups.**

| **Follow-up** | **AVR Group** | | **MVR Group** | |
| --- | --- | --- | --- | --- |
|  | **PCS** | **MCS** | **PCS** | **MCS** |
| Baseline | 33.8±7.4 | 43.4±9.6 | 32.1±6.4 | 43.5±10.2 |
| 1-month | 37.1±5.5 | 47.5±8.6 | 39.7±6.5 | 45.2±8.3 |
| 6-month | 43.4±6.7 | 52.7±7.5 | 44.5±6.4 | 48.5±8.4 |
| 1-year | 45.2±7.4 | 53.0±7.4 | 43.4±8.6 | 52.2±6.8 |
| 2-year | 47.1±6.2 | 55.3±5.9 | 48.2±7.9 | 53.3±7.0 |
| 3-year | 49.0±7.0 | 54.9±7.8 | 50.2±7.4 | 53.8±7.4 |
| p-value (Baseline *vs.* 3-year) | <0.001 | <0.001 | <0.001 | <0.001 |

MCS: Mental component summary scores; PCS: Physical component summary scores.

**Supplementary Table S2. Hemodynamic data of patients with Small Aortic Annulus (n=55).**

| **Echo parameter** | **Baseline** | **Discharge** | **1-month** | **6-month** | **1-year** | **2-year** | **3-year** |
| --- | --- | --- | --- | --- | --- | --- | --- |
| **19 mm group** | | | | | | | |
| Peak Pressure Gradient (mmHg) | 85.9±35.9 | 29.0±12.3 | 25.8±7.1 | 25.1±7.2 | 28.4±10.3 | 27.6±10.4 | 24.3±13.7 |
| Mean Pressure Gradient (mmHg) | 54.3±25.1 | 15.4±6.7 | 13.8±4.3 | 13.8±4.3 | 16.3±7.1 | 14.7±5.3 | 12.5±6.3 |
| EOA (cm^2^) | 0.8±0.3 | 1.5±0.5 | 1.4±0.4 | 1.6±0.4 | 1.5±0.4 | 1.4±0.3 | 1.6±0.5 |
| IEOA (cm^2^/m^2^) | 0.5±0.2 | 0.9±0.3 | 0.9±0.2 | 1.0±0.3 | 1.0±0.3 | 0.9±0.2 | 1.0±0.3 |
| **21 mm group** | | | | | | | |
| Peak Pressure Gradient (mmHg) | 87.7±26.0 | 27.1±17.0 | 22.5±4.9 | 22.2±6.4 | 22.8±8.2 | 20.1±6.7 | 20.5±12.3 |
| Mean Pressure Gradient (mmHg) | 56.4±19.7 | 14.9±9.0 | 12.0±2.8 | 12.1±4.4 | 12.8±5.4 | 10.7±4.4 | 10.7±6.5 |
| EOA (cm^2^) | 0.8±0.3 | 1.6±0.6 | 1.7±0.3 | 1.7±0.4 | 1.7±0.4 | 1.8±0.4 | 1.9±0.3 |
| IEOA (cm^2^/m^2^) | 0.5±0.2 | 1.0±0.4 | 1.0±0.2 | 1.0±0.3 | 1.0±0.2 | 1.0±0.2 | 1.1±0.4 |

**Supplementary Table S3. NYHA Functional improvement of patients with Small Aortic Annulus (n=55).**

| **NYHA Functional Class of Patients Implanted with 19-mm size (n=29)** | | | | | | | |
| --- | --- | --- | --- | --- | --- | --- | --- |
| **NYHA Class** | **Baseline**  **(n=29)** | **Discharge**  **(n=29)** | **1-month**  **(n=29)** | **6-month**  **(n=29)** | **1-year**  **(n=29)** | **2-year**  **(n=28)** | **3-year**  **(n=25)** |
| Class I | 2 (6.9) | 22 (75.9) | 28 (96.6) | 28 (96.6) | 28 (96.6) | 28 (100.0) | 22 (88.0) |
| Class II | 5 (17.2) | 7 (24.1) | 1 (3.5) | 1 (3.5) | 1 (3.5) | 0 (0.0) | 2 (8.0) |
| Class III | 22 (75.9) | 0 (0.0) | 0 (0.0) | 0 (0.0) | 0 (0.0) | 0 (0.0) | 1 (4.0) |
| Class IV | 0 (0.0) | 0 (0.0) | 0 (0.0) | 0 (0.0) | 0 (0.0) | 0 (0.0) | 0 (0.0) |
| **NYHA Functional Class of Patients Implanted with 21-mm size (n=26)** | | | | | | | |
| **NYHA Class** | **Baseline**  **(n=26)** | **Discharge**  **(n=25)** | **1-month**  **(n=24)** | **6-month (n=24)** | **1-year**  **(n=24)** | **2-year**  **(n=24)** | **3-year**  **(n=24)** |
| Class I | 1 (3.9) | 19 (76.0) | 22 (91.7) | 23 (95.8) | 23 (95.8) | 22 (91.7) | 23 (95.8) |
| Class II | 5 (19.2) | 4 (16.0) | 2 (8.3) | 1 (4.2) | 1 (4.2) | 2 (8.3) | 1 (4.2) |
| Class III | 20 (76.9) | 2 (8.0) | 0 (0.0) | 0 (0.0) | 0 (0.0) | 0 (0.0) | 0 (0.0) |
| Class IV | 0 (0.0) | 0 (0.0) | 0 (0.0) | 0 (0.0) | 0 (0.0) | 0 (0.0) | 0 (0.0) |

**Supplementary Table S4. Improvements in the Mean SF-12 scores of patients with Small Aortic Annulus.**

| **Follow-up** | **19 mm (n=29)** | | **21 mm (n=26)** | |
| --- | --- | --- | --- | --- |
|  | **PCS** | **MCS** | **PCS** | **MCS** |
| Baseline | 35.1±8.1 | 43.6±9.8 | 32.6±5.7 | 41.6±8.6 |
| 1-month | 36.6±5.0 | 47.5±8.7 | 36.6±5.3 | 46.6±8.8 |
| 6-month | 44.9±6.8 | 55.1±5.0 | 42.9±6.1 | 50.5±7.7 |
| 1-year | 46.6±6.4 | 53.9±6.8 | 46.2±7.6 | 52.5±7.3 |
| 2-year | 47.4±5.0 | 55.3±6.2 | 46.8±6.5 | 54.3±6.4 |
| 3-year | 47.6±7.8 | 55.3±7.2 | 49.8±5.9 | 54.1±8.2 |

MCS: Mental component summary scores; PCS: Physical component summary scores.

**Supplementary Table S5: Cumulative 3-year Clinical Outcomes of Patients with Small Aortic Annulus**

| **Events, n (%)** | **19-mm**  **(n=29)** | **21-mm**  **(n=26)** | **Cumulative Number of Events** | **% events per 100 patient-years** |
| --- | --- | --- | --- | --- |
| All-cause mortality | 1 | 2 | 3 | 1 |
| Myocardial infarction | 0 | 0 | 0 | 0 |
| Stroke | 2 | 0 | 2 | 0.67 |
| MACE | 3 | 2 | 5 | 1.67 |
| Major & minor bleeding | 0 | 0 | 0 | 0 |
| AKI | 0 | 0 | 0 | 0 |
| Valve thrombosis | 0 | 0 | 0 | 0 |
| SVD | 0 | 0 | 0 | 0 |
| Repeat hospitalization | 3 | 3 | 6 | 2 |
| Conduction disturbances and arrhythmias/PPI | 0 | 0 | 0 | 0 |
| Explant | 0 | 0 | 0 | 0 |
| Haemolysis | 0 | 0 | 0 | 0 |
| Valve related re-operation | 0 | 0 | 0 | 0 |

Total follow-up: 164 patient-years. MACE: MACE is defined as a composite of all-cause mortality, myocardial infarction, and stroke. AKI: Acute kidney injury; PPI: Permanent pacemaker implantation; MACE: Major adverse cardiovascular event; SVD: Structural valve deterioration.
